# Exploration of the Association between Nurses' Moral Distress and Secondary Traumatic Stress Syndrome: Implications for Patient Safety in Mental Health Services

**DOI:** 10.1155/2017/1908712

**Published:** 2017-10-25

**Authors:** Maria Christodoulou-Fella, Nicos Middleton, Elizabeth D. E. Papathanassoglou, Maria N. K. Karanikola

**Affiliations:** ^1^Mental Health Services, Ministry of Health, Nicosia, Cyprus; ^2^Department of Nursing, School of Health Sciences, Cyprus University of Technology, No. 15, Vragadinou str, 3041 Limassol, Cyprus; ^3^Faculty of Nursing, 5-262 Edmonton Clinic Health Academy (ECHA), University of Alberta, 11405-87th Ave., Edmonton, AB, Canada T6G 1C9

## Abstract

Work-related moral distress (MD) and secondary traumatic stress syndrome (STSS) may be associated with compromised health status among health professionals, reduced productivity, and inadequate safety of care. We explored the association of MD with the severity of STSS symptoms, along with the mediating role of mental distress symptoms. Associations with emotional exhaustion and professional satisfaction were also assessed. This cross-sectional survey conducted in 206 mental health nurses (MHNs) was employed across public sector community and hospital settings in Cyprus. The analysis revealed that MD (measured by the modified Moral Distress Scale) was positively associated with both STSS (measured by the Secondary Traumatic Stress Scale) and mental distress symptoms (assessed by the General Health Questionnaire-28). The association of MD with STSS symptoms was partially mediated by mental distress symptoms. This association remained largely unchanged after adjusting for gender, age, education, rank, and intention to quit the job. Our findings provide preliminary evidence on the association between MD and STSS symptomatology in MHNs. Situations that may lead health professionals to be in moral distress seem to be mainly related to the work environment; thus interventions related to organizational empowerment of MHNs need to be developed.

## 1. Introduction

Healthcare organizations are not simply institutes which provide care; they are also workplaces for over 59 million workers worldwide [1]. People employed in healthcare services are exposed daily to a complex variety of health and safety hazards which include psychosocial risks, such as those associated with work-related stress [1]. Nursing personnel is the largest group of health professionals in healthcare systems. Thus, nurses are often on the epicenter of empirical research on work-related stress, which has been associated not only with substandard quality and safety of care [2, 3], but also with poor health status, decreased quality of life, and compromised safety among personnel [4–8].

Over a decade ago, the Word Health Organization, recognizing work-related risks as a public health priority, stated the need for better management of work-related risks for healthcare workers [9]. Additionally, evidence shows that the protection of mental and physiological well-being of healthcare workers contributes to increased quality and safety of patient care as well as to the sustainability of healthcare systems. 

The 2006 World Health Report “Working Together for Health” on human resources mentioned a global shortage of healthcare employees, mainly nurses, which had reached crisis levels in 57 countries, including the Eastern Mediterranean region [9]. As a response, during the last decade, there has been increased focus on ways to decrease the severity and adverse impact of work-related risks on nurses and physicians in order to improve staff retention and prevent turnover.

### 1.1. Literature Review

Over the last decade, a body of evidence accumulated from research in occupational medicine and organizational psychology indicates new and broader sources of work-related stress for nurses, such as moral distress [10, 11]; severe degree of professional burnout [4, 12, 13]; and increasing mental health problems associated with workplace originated traumas, such as secondary traumatic stress syndrome symptoms [14–18].

Although both, moral distress and secondary traumatic stress syndrome, have been identified as occupational hazards for health professionals providing care to vulnerable populations, that is, consumers of Mental Health Services, little is known about the association between them [10, 14], as well as their link with work-related adverse phenomena (i.e., low job satisfaction, professional burnout, intention to resign, and absence of empathy), especially more so in highly stressful work environments such as Mental Health Services [16, 19–22]. Additionally, although both syndromes have similar manifestations, for example, physiological arousal, functional impairment, and distressing emotions, there are scant data in mental health nurses about the association with mental distress, as defined by compromised mental health status expressed by general symptoms of mental disturbance, for example, anxiety and depressive symptoms [10, 14, 18].

In more detail, moral distress arises when one must act in a way that contradicts his/her personal beliefs and values [10]. Thus, moral distress manifests as suffering experienced by health professionals when they know which is the right moral decision; however, they are unable to implement this decision due to a multitude of factors that might relate to their coworkers or the norms and practices of the institution [23]. Moral distress in nurses has been associated with psychological discomfort and low patient safety standards, for example, dysfunctional communication among clinicians, medication errors, and dysfunctional work attitudes, including burnout, intention to quit the work, and low job satisfaction [10, 11, 20]. Job satisfaction reflects one's positive emotions in relation to her/his occupation [24]. A central dimension of health professionals' job satisfaction is satisfaction from therapeutic relationships, which reflects the amount of positive feelings experienced by clinicians in relation to the therapeutic encounter with consumers of healthcare services [24]. 

Secondary traumatic stress syndrome (STSS) develops in health professionals who come into continuous and close contact with trauma survivors, while experiencing considerable emotional disruption themselves, thus becoming indirect victims of the trauma, they care for [21, 22]. To date, there has been evidence from cross-sectional studies in nurses which associate STSS with mental and somatic distress symptoms, low job satisfaction, and burnout [25–27]; however, these data are limited in mental health nurses.

On the other hand, professional burnout involves a state of emotional, physiological, and personal esteem distress resulting from exposure to prolonged work-related stressful conditions [28]. The core concept of professional burnout is emotional exhaustion, which regards experiences of psychological fatigue related to work conditions, while in this state one's drive is replaced by tiredness, commitment by cynic behaviour, and efficiency by low motivation [28]. Experiences of emotional exhaustion in nurses have been linked with both moral distress [29] and STSS [25], as well as reduced productivity, low job satisfaction, high rates of resignations and turnover [4], low patient safety standards [2, 20], and mental distress, for example, anxiety and depressive symptoms [30].

Anxiety and depressive symptoms in nurses are also associated with adverse work behaviours, such as avoidance, increased irritability, and temper or cynical attitude [31, 32], as well as high percentages of mistakes during medication administration [33]. Additionally, both mental and physical distress in nurses are related to increased percentages of sick leaves [34], resignations, turnover, or even discouragement of young people to follow this profession [35]. Furthermore, not only is nurses' mental distress related to low patient safety standards and reduced productivity but it may also lead to health system burden, as far as financial and human resources are concerned [2, 20, 33]. 

Additionally, nurses' mental distress and psychological discomfort, that is, emotional exhaustion, STSS symptoms, low job satisfaction, and moral distress, seem to be associated with impersonal attitude towards patients, indifference, and anger manifestations [12, 36, 37], negatively influencing the quality of interaction with the consumers of health services and colleagues [23, 27, 38]. Yet, the effectiveness of the therapeutic relationship constitutes a fundamental element of the quality of care provision, as well as patient safety standards [39–41], while empathy is a core clinical skill towards this [42]. Empathy is a cognitive process, regarding clinicians' ability to understand the unique experiences of others, for example, patients and their families, and communicate their understanding with them [40–42]. Despite the importance of nurses' empathy and satisfaction from therapeutic relationships in clinical settings, studies related to this issue are limited, particularly their association with work-related stressors and manifestation of mild psychiatric symptoms [38, 41].

Taking into consideration the aforementioned evidence, the exploration and subsequent effective management of both work-related stress and potentially mental distress in nurses are considered highly important [18]. In this context, the aim of this study was to explore the association of the intensity and frequency of morally distressing situations with symptom severity of secondary traumatic stress syndrome, the potentially mediating effect of mental distress in this association, and possible associations with work-related adverse attitudes, among mental health nurses in Cyprus.

### 1.2. The Context of Mental Health Services

Nurses employed in Mental Health Services (MHN) may encounter a series of ethical issues and related problems, which may lead to morally distressing experiences [43, 44]. Moral distress is a phenomenon of increasing concern in mental health nursing practice, education, and research due to the increased frequency of ethically and morally charged situations in mental healthcare settings compared to other clinical contexts [22, 45, 46]. The psychopathological changes in people suffering from mental illness may undermine their capacity to consent treatment procedures [47]. The consequences may range from nonparticipation in clinical decision-making to restrictive measures for risk prevention and treatment, such as involuntary admissions, involuntary intramuscular medication, or physical restraint [48]. Informed consent for treatment encompasses information giving, comprehension, and volunteerism; thus these situations may be viewed as violation of the main bioethical principles in healthcare treatment, that is, autonomy, nonmaleficence, beneficence, and justice causing ethical and subsequent psychological discomfort to clinicians [49]. Additionally, neurocognitive dysfunction in people experiencing mental illness, for example, disturbed thought processes, hallucinations, or illusions, may jeopardize their critical thinking capacity and render them vulnerable to violent or deceptive behaviours, even healthcare workers, arising morally stressful issues in MHNs who are endorsed to advocate for patients and their families [50]. In other cases, MHNs may judge that the administration of pharmacological restrictive measures is not the most appropriate approach for an irritated patient; however they may follow the expectation to implement it according to physician's orders [51]. In such cases, MHNs may feel morally burdened, even though they may not be able to change what is happening [48, 52, 53]. Overall, morally distressing experiences may have a traumatic effect on the physical and mental health of healthcare professionals [10], since MHNs suffering from moral distress describe feelings of frustration, anger, and guilt [51], usually expressed as general symptoms of mental distress, for example, anxiety and depressive symptoms [24].

In addition, people suffering from mental illness very often describe themselves, after being exposed to traumatic experiences, as trauma survivors [50]. Since secondary traumatic stress syndrome concerns the prolonged effects of a traumatic event to those who care for trauma survivors, one may argue that those who care for the mentally ill may also experience the indirect effect of these traumatic experiences [24].

Apart from morally distressful situations and secondary traumatic stress syndrome, emotional exhaustion has also been identified as a job-related hazard for MHNs. Studies in MHNs have reported association between emotional exhaustion and several health outcomes, including low self-reported somatic health level [54, 55], anxiety and depressive symptoms [56, 57], or higher risk of health-threatening behaviours, such as tobacco smoking [58] or consumption of alcohol [51].

The main work-related stressors encountered by MHNs, possibly associated with psychosocial and job-related hazards, may be grouped into two categories [36, 51, 55, 59, 60]: those stressors related to the therapeutic relationship and the nursing process of care, namely, challenging behaviours by patients and their family members, physical and verbal violent behaviour towards clinicians by patients, patients' suicidal behaviour, nonadherence to therapy, and insufficient time to provide optimal care, and those related to the working context, that is, poor staffing, inadequate referral systems, heavy workload, insufficient resources, type of working setting (community versus hospital settings), and uncertainty of employment.

The aforementioned stressors have been associated with emotional exhaustion, intention to quit the job, low job satisfaction, and diminished engagement in the therapeutic process in mental health nursing populations. The latter may be expressed by absence of empathy [36, 41, 42, 54, 56]. At the same time, the above stressors may trigger morally distressing experiences in MHNs, as well [51–53, 60]. Data, mostly from qualitative studies, show that, due to heavy workload and time pressure linked with insufficient staffing, MHNs very often are not able to develop a therapeutic relationship and effective communication with patients, while in other cases healthcare professionals may keep the restriction measures, even if it is not necessary due to these reasons. Yet, these findings may support possible explanations regarding how stressful work conditions and relevant circumstances may be linked not only with emotional exhaustion, but also with morally and ethically disturbing experiences.

## 2. Aim

The aim of the present study was to explore, among mental health nurses in Cyprus, the following: (a) the frequency and intensity of morally distressing (MD) situations, (b) the severity of symptoms of secondary traumatic stress syndrome (STSS) and mental distress, as well as the degree of emotional exhaustion and job satisfaction, (c) the association among MD, STSS, and mental distress symptoms, (d) the association of MD and STSS symptoms with sociodemographic factors and work-related features, including job satisfaction, satisfaction from therapeutic relations, emotional exhaustion, and empathy, and (e) the extent to which self-rated degree of general mental distress symptoms mediates the association between MD and STSS symptoms.

In order to address the aforementioned objectives, the following research questions were formulated:

- What is the frequency and intensity of morally distressing experiences?
- What is the degree of (i) symptoms of STSS, (ii) symptoms of mental distress, (iii) empathy, (iv) emotional exhaustion, and (v) job satisfaction?
- Is there any association between MD measures (i.e., intensity and frequency) and the severity of symptoms of (i) mental distress and (ii) STSS?
- Is there any association between MD measures and (i) sociodemographic factors and (ii) work-related factors, that is, job satisfaction and satisfaction from therapeutic relations, emotional exhaustion, and empathy?
- Does self-rated degree of general mental distress symptoms mediate the association between MD measures and the severity of STSS symptoms?

## 3. Materials and Methods

### 3.1. Study Design and Setting

The study was designed as a cross-sectional descriptive correlational study with the use of self-reported questionnaire scales. After the reform following deinstitutionalization in Europe and the USA, Mental Health Services in Cyprus are provided in a wide range of settings, including both hospital and community-based services. The latter are provided either at home or in community centers (child and adolescent Mental Health Services, day hospitals, community mental health centers, and substance abuse and addiction treatment programs). Hospital-based services include acute services in the psychiatric clinics of the general hospitals and institutional services in Athalassa Psychiatric Hospital, which is Cyprus' main psychiatric hospital.

### 3.2. Ethical Issues

The study protocol was approved by the Cypriot National Bioethics Committee (PN: CNBV/EP/2015/11) and the Research Promotion Committee of the Ministry of Health (PN: 5.34.01.7.2E). Moreover, the Commissioner for Personal Data Protection was notified as required (PN: 3.28.325). The questionnaire packet included an information sheet explaining the aim of the study and the voluntary nature of participation. Participants signed an informed consent form. Participation was anonymous. Names or any other personal information which could reveal the identity of the respondents was not reported at any part of the questionnaire. Confidentiality was guaranteed and the completed questionnaires were returned in nontransparent sealed envelopes. Permissions were obtained for the use of all copyrighted questionnaire scales.

### 3.3. Eligibility and Sample

The target population consisted of all registered MHNs employed in the public sector either in child/adolescent or in adult Mental Health Services, in any of the aforestated settings (*N* = 360). Eligibility criteria included employment in a clinical setting for at least six months. Mental health nurses with less than 6 months' work experience or seconded to nonclinical settings were excluded. While power analysis indicated that a sample of 100 participants would provide sufficient power (90%) for a statistically significant (alpha = 0.05) correlation in the magnitude of 0.3 between the main study variables, consistent with previous literature estimates [16]; the minimum sample size was set to 200 in order to allow the exploration of the metric properties of the measurement scales as well [61].

### 3.4. Data Collection Procedure

A total of 360 questionnaire packets along with consent forms were distributed to all eligible members of the target population by personal visits of the main researcher (MC). The participants were asked to respond to the questionnaire at their own time and return it in a sealed envelope by placing it in a box located at each work setting for the purpose of the study. After 2 weeks, a reminder was given and the boxes were collected by the end of the third week. The final sample consisted of 206 completed questionnaires (response rate 57.2%).

### 3.5. Instruments

The self-reported questionnaire pack consisted of two parts: (i) demographic, educational, personal, and work-related information and (ii) structured scales: modified version of Moral Distress Scale for Mental Health Services (M-MDS-MHS); Secondary Traumatic Stress Scale (STSS); General Health Questionnaire-28 (GHQ-28); and Jefferson Empathy Scale (JES).

#### 3.5.1. Part I

*Sociodemographic and Personal Data*. Two groups of sociodemographic data were collected: work history data (years of nursing experience, years in the current position, type of work setting, and rank) and personal variables (age, gender, marital status, number of children, and education). Since international literature indicates association between work-related dysfunctional attitudes and health-threatening behaviours [51, 57, 58], the following variables were also included in the personal data questionnaire: “How many times in a week do you drink alcohol?” and “How many times in a week do you exercise?” Additionally, since international literature indicates association between work-related attitudes and self-perceived social and personal life satisfaction [51, 57–59], the following variables were also included in the personal data questionnaire with a numeric rating scale (NRS) response formulation: “Please, indicate from 1 to 10 how satisfied you feel with your: (a) social life and (b) personal life.” The range of the responses in these questions was from 1: not at all satisfied to 10: completely satisfied.

In relation to work-related variables, the participants also filled in a short questionnaire encompassing staff safety measures, reflecting MHNs' workload. The information given was about nurse-per-patient ratios and number of patients treated in the setting. Nurses' workload has been previously associated with emotional exhaustion and mental and moral distress [2, 11, 25]. Additionally, a question was included regarding the intention to leave the current post, or having quit a previous clinical post due to distress related to care or patient-related decisions, since this factor has been associated elsewhere with the main variables explored herein [2, 11, 25].

*Job Satisfaction and Burnout Measures*. Finally, a series of questions regarding work-related attitudes were included with a numeric rating scale (NRS): 1–10. The first item regarded the (i) degree of emotional exhaustion, as the core concept of professional burnout. This question was formulated as follows: “Please, indicate from 1 to 10 how emotionally exhausted do you feel due to your work,” while the range of the responses in this question was from 1: not at all exhausted to 10: completely exhausted. The second question assessed the degree of satisfaction from (a) the profession and (b) therapeutic relations. The formulation and response range of the two latter questions was like the emotional exhaustion question; that is, “Please, indicate from 1 to 10 your level of professional satisfaction with regard to (a) your work as MHN, (b) therapeutic relations with patients and their families” (1: not at all satisfied to 10: completely satisfied).

#### 3.5.2. Part II

*Moral Distress Assessment Instrument. *The frequency and intensity of morally distressing experiences were assessed with a modified version of the Moral Distress Scale-Revised (MDS-R), Adult Nurse Version, that is, M-MDS-MHS. The M-MDS-MHS used in this study was largely based on the Moral Distress Scale (MDS-R) by Hamric et al. [62] after permission was obtained by the developers of the original (Professor Corley) and the revised scale for nurses caring for adults (Professor Hamric). The M-MDS-MHS used herein was adapted accordingly for psychiatric clinical settings based on the Moral Distress Scale developed by Ohnishi et al. [60] and the MDS-R. Specifically, since five items (1, 4, 5, 11, and 16) of the Ohnishi et al. [60] scale were common to the MDS-R scale, it was deemed necessary to integrate the remaining ten items of the Ohnishi et al. [60] scale to the MDS-R version. Furthermore, item 7 of the MDS-R was eliminated since it was not applicable to Mental Health Services context. The modified scale was translated into Greek using the forward-backward method. The face and content validity of the modified scale were assessed by a panel of five experts. The process resulted in a slight modification in the wording of a number of items to correspond more effectively to the Mental Health Services context, as well as the addition of two items. The final scale consisted of 32 items. The reasons and process for developing the modified version of the scale is described in detail elsewhere [51].

Briefly, the MDS-R scale developed by Hamric et al. [62] was designed for acute settings, medical surgical, thus not specific for mental health settings. As a result, organizational or cultural conditions prevailing in mental health settings may be underestimated by this tool. Similarly, although the instrument developed by Ohnishi et al. [60] was constructed for Mental Health Services, it was however oriented for hospital, mainly, institutional environments. Japanese Mental Health Services have not followed deinstitutionalization reformation. Consequently, the instrument has limited applicability to healthcare systems of different orientation, that is, in European or North American settings.

The M-MDS-MHS used herein encompasses two parts, each of which includes the same 32 items. In the first part, the frequency of morally distressing incidences is measured on a 5-point Likert scale ranging from 0 (never) to 4 (very frequently), with an overall score ranging from 0 to 128. In the second part, participants rate the intensity of disturbance felt when experiencing each distressing situation listed in the first part of the instrument on a 5-point Likert scale ranging from 0 (none) to 4 (great extent). The overall score on this part of the scale also ranges between 0 and 128. Additionally, in order to measure the overall severity of moral distress the score from each item in the first part is multiplied by the corresponding score in the second part, leading to a composite score reflecting both the frequency and the intensity of the distressing experiences. For each item, the score can range between 0 and 16. The theoretical range of the “composite moral distress score” across all 32 items is 0 to 512. The higher the score the higher the severity of the experienced moral distress. Examples of the items included in the M-MDS-MHS are as follows: “Work with levels of nurse or other care provider staffing that I consider unsafe” or “Avoid taking action when I learn that a physician or nurse colleague has made a medical error and does not report it” or “I secretly mix medication into patients' food or drink when they have refused it.” The metric properties of the M-MDS-MHS were assessed. Internal consistency reliability assessed by Cronbach's alpha coefficient was 0.893 for the frequency MD scale and 0.941 for the intensity MD scale. Construct validity was measured via factor analysis with varimax rotation. The unidimensionality of the scale was confirmed, explaining the 31.64% of the variance for the intensity subscale and 30.30 for the intensity subscale.

*STSS Symptoms Assessment Instrument*. The degree of symptoms of secondary traumatic stress syndrome was assessed using the Secondary Traumatic Stress Scale developed by Bride et al. [63]. This comprises 17 items constructed according to the symptoms of posttraumatic stress disorder described in the Diagnostic and Statistical Manual for Psychiatric Disorders-IV-Text Revision [32]. Each item is rated on a 5-point Likert scale, from 1 (never) to 5 (very often). In particular, respondents are asked to report the frequency to which they have experienced the symptoms described in each item during the previous two weeks. Examples of the items are “I was less active than usual” or “I had trouble to concentrate.” The scores can range from 17 to 85, with higher scores indicating more severe self-reported symptoms of secondary traumatic stress syndrome. The items of the scale address intrusion symptoms, avoidance symptoms, and symptoms of arousal, reflecting the three subscales of the tool. Internal consistency reliability assessed by Cronbach's alpha coefficient has been reported previously as 0.93 for the entire scale [63]. The validity of the scale has also been assessed elsewhere [63] via confirmatory factor analysis using structural equation modeling, providing values representing adequate model fit; that is, GFI = 0.90, CFI = 0.94, IFI = 0.94, and RMSEA = 0.069. Moreover, this procedure resulted in the confirmation of the three factors included in the scale, identified as intrusion, avoidance, and arousal [63]. Although this scale may be applied as a three-dimension tool, it is also applicable in the unidimension version, as it was used herein [63].

*Empathy Assessment Instrument. *The degree of empathy among nurses was assessed by the Jefferson Scale of Empathy-HP-Version [42]. This is a 20-item scale, rating nurses' feelings and perceptions of nurse-patient relationship on a 7-point Likert scale. The responses to each item range from 1 (strongly disagree) to 7 (strongly agree), and the total score can range between 20 and 140. Internal consistency reliability assessed by Cronbach's alpha coefficient has been reported previously as 0.81 [41]. Additionally, the construct validity has been assessed elsewhere via factor analysis with varimax rotation. This procedure resulted in the confirmation of the three factors included in the scale, identified as “Perspective Taking”; “Compassionate Care”; and “Standing in Patients' Shoes” [41]. Although this scale may be applied as a three-dimension tool, it is also applicable in the unidimension version according to the constructors of the scale, as it was used herein [41, 42]. Examples of the items included in the scale are as follows: “My patients feel better when I understand their feelings” or “I try to imagine myself in my patients' shoes when providing care to them.”

*General Symptoms of Mental Distress Assessment Instrument*. The degree of general symptoms of mental distress was assessed by the Greek version of the General Health Questionnaire [64]. This is a widely used questionnaire of 28 items, nonspecific measure of mental distress, often used as a screening tool for depressive and relevant anxiety symptoms in both general population and clinical samples. The responses in this instrument are given in a 4-point Likert scale [(0): never to (3): more than usual] divided into 4 subscales, reflecting the four dimensions of the tool: general health disturbance symptoms (somatic symptoms) (7 items); anxiety symptoms (7 items); self-perception of personal/social functioning (7 items); and depressive symptoms and suicidal behaviour (7 items) [64, 65]. The metric properties of the GHQ-28 have been explored in numerous studies [64, 65]. Values of Cronbach's alpha ranging from 0.80 to 0.92 regarding internal consistency reliability have been reported for all the four subscales and the entire scale elsewhere [64, 65]. Examples of the items are “Have you recently felt ill/constantly under strain/that life is not worth living?”

### 3.6. Data Analysis

Descriptive statistics of all sociodemographic and other variables were calculated using frequencies or mean (*M*) and standard deviations (SD) for categorical and continuous variables, respectively. In the absence of accepted cut-off values for all structured instruments applied herein, we used the quartiles of the theoretical range of each scale in order to interpret relevant scores as low, moderate, or high. Additionally, since all instruments were used for the first time in Cypriot MHN, the study assessed their metric properties. The dimensionality and construct validity of the scales were assessed in exploratory factor analysis with principal component extraction and varimax rotation (reported elsewhere) [51]. The internal consistency of the overall scale and subscales was estimated using Cronbach's alpha coefficient. Τest-retest reliability was assessed in a subsample of 20 MHNs completing all scales twice, with one week apart, using Kendal's tau. This procedure was followed to assess the stability of the instruments (temporal reliability). Internal consistency and test-retest reliability values are presented in the Results. The overall and subscale scores for all study variables were calculated as the sum of item responses, according to the constructors of the instruments [42, 63, 64], and normality checks were performed. Differences in study variables by sociodemographic and other characteristics of the participants were assessed using parametric tests (*t*-test and one-way ANOVA) as appropriate. The correlation between the main study variables was assessed by calculating the Pearson correlation coefficient. Finally, the association between MD as indexed by the composite score (independent) and STSS score (dependent variable) was assessed in linear regression models before and after adjusting for (a) general symptoms of mental distress as measured by the GHQ-28 (mediator) and for (b) other sociodemographic and work-related characteristics (covariates). Data analysis was performed in Statistical Package for Social Sciences ver. 21.00.

## 4. Results

### 4.1. Sociodemographics, Work-Related Characteristics, Self-Reported Satisfaction Measures, and Related Associations

The final sample consisted of 206 participants, mean age 35.3 years [standard deviation (SD) = 7.6], with 11.6 years of work experience on average in Mental Health Services (SD = 7.4), of whom 43.7% were men and 56.3% were women. This is largely consistent with the expected gender proportions among mental health nurses in Cyprus based on official statistics (48.3% and 51.6%, for males and females, resp.). One in three held a postgraduate degree (35.9%). The observed distribution in the sample, in terms of the different geographical regions as well as settings employed, was largely representative of the expected proportions.  Table 1 presents the sociodemographic and work-related characteristics and attitudes of the participants, as well as their self-rated frequency of life-threatening behaviours and degree of satisfaction with personal and professional life. While participants rated satisfaction from personal life (*M* = 8.0, SD = 1.5) and social relationships (*M* = 7.9, SD-1.4) quite high on a NAS 0–10, they rated satisfaction from work quite low by comparison (*M* = 6.6, SD 2.1). On average, participants reported a moderate degree of job-related emotional exhaustion (*M* = 4.8, SD = 2.7), while as many as one in four reported that they considered leaving their current position. Based on the responses of 126–130 participants who provided information with regard to staff safety measures number of patients treated in the setting (*M* = 9.7, SD = 5.9, range 2–40) and nurse-to-patient ratios (*M* = 7.6, SD = 9.9, range 1–40) were reported.

### 4.2. Summary Statistics for the Main Study Variables

Table 2 presents descriptive statistics for the main study variables along with Cronbach's alpha coefficient for internal consistency and test-rest correlation for the corresponding measurements scales, which were satisfactory in all cases.

#### 4.2.1. Frequency and Intensity of Morally Distressing Experiences

The observed distributions of overall scores of the frequency as well as the intensity of moral distress were fairly symmetrical with *M* = 37.8 (median = 38.0, SD = 20.1, range: 1–103, and IQR: 24–51) and *M* = 70.4 (median = 71.0, SD = 32.1, range: 0–127, and IQR: 47–97), respectively. Using the quartiles of the theoretical range of the scale (0–128) in the absence of accepted cut-off values, the frequency of morally distressful incidences would be characterized as low to moderate (i.e., in the lower range of the second quartile: 33–65). 41.3% of the participants had scores in the lower quartile of the theoretical range of the scale (<32), reporting never or rarely experiencing any of the 32 potentially morally distressful situations. Thus, the remaining participants (58.7%) reported that they experienced at least some of the 32 situations described on the scale relatively more frequently, and, in fact, as many as 47.6% reported experiencing at least one of these situations very often. Furthermore, the intensity of moral distress from these incidences, irrespective of their frequency, is moderate to high (i.e., in the third quartile 66–96). As many as 26.2% of the participants had scores higher than 96 (top quartile of theoretical range) on the moral distress intensity scale. In terms of the various potentially morally distressful situations,* “assisting a doctor or/and a nurse, who according to my opinion does not have the appropriate skills to provide care” *was by far the most frequently reported, with as many as 49.5% of the participants responding that this happens often or very often (M = 2.3, SD = 1.3)—results not shown in detail. This was followed by the statement﻿* “I undertake extensive therapeutic interventions following medical instructions even when I do not think they will change the clinical picture of the patient,”* reporting that they occur often or very often by 35% of the participants (*M* = 1.9, SD = 1.2). Nevertheless, these were not the situations which were rated as the more intense in terms of moral distress. In descending order, the top three single items with the highest moral distress intensity scores were as follows: “*ignore situations where there is a suspicion that one of the patients is being badly treated or abused by some of the healthcare staff*,” with 62.6% rating it as intense or very intense, even though 7.3% reported that it occurs often or very often; “*assist doctors in conducting examinations or treatments without the informed consent of the patient,”* with 45.1% intense/very intense and 11.6% in terms of its frequency (often or very often); and “*the nurse-to-patient ratio is not safe,”* with 52% prevalence of intense/very intense and 6.3% in terms of its frequency (often or very often).

#### 4.2.2. Degree of Secondary Traumatic Stress Syndrome Symptoms, Empathy, and General Mental Distress Symptoms

In terms of the other main study variables, the mean score and standard deviations were as follows: *M* = 31.1 (SD = 10.2) on the STSS scale (indicating moderate to low degree of symptoms of secondary traumatic stress syndrome on average) and *M* = 105.9 (SD = 12.7) on the Jefferson Scale for Empathy (indicating moderate to high levels of empathy) and *M* = 19.4 (10.0) on the GHQ-28 (indicating moderate to high levels of general mental distress symptoms). As many as 25.7% of the participants had scores higher than the lower range of the 2nd quartile of the theoretical range of the scale, indicating clinically relevant symptoms, with higher scores on average in descending order, in terms of personal/social dysfunction, somatic symptoms, anxiety, and depressive symptoms subscales.

### 4.3. Associations between MD Measures and Main Study Variables

Table 3 presents the correlations between the main study variables. A moderate positive correlation was observed between the composite score of MD (frequency × intensity) and STSS scale score (*r* = 0.35; *p* value < 0.001). Composite moral distress score also showed a moderate positive correlation to general mental distress symptoms, as measured by the GHQ-28 (*r* = 0.29; *p* value < 0.001), with higher correlations with somatic symptoms and anxiety/insomnia subscales. Furthermore, the GHQ-28 score had a strong positive correlation (*r* = 0.65; *p* < 0.001) with the STSS score, with the strongest correlation observed with the anxiety/insomnia subscale (*r* = 0.70; *p* < 0.001), followed by somatic symptoms (*r* = 0.52; *p* < 0.001), and the weakest correlation with self-perceived personal/social dysfunction subscale (*r* = 0.37; *p* < 0.001). Finally, in terms of empathy as measured by Jefferson Scale of Empathy, with the exception of a small positive correlation with the intensity of moral distress (*r* = 0.18) and a negative correlation with depressive symptoms (*r* = −0.21), no other significant correlations were observed between empathy scores and the main study variables.

There were mild statistically significant correlations between composite moral distress score and (a) satisfaction from therapeutic relations (*r* = −0.21; *p* < 0.010), (b) degree of emotional exhaustion (*r* = 0.19; *p* = 0.01), and (c) job satisfaction (*r* = −0.15; *p* = 0.03), but there was no association with general life satisfaction and satisfaction from social relations—see Table 4. In descending order, degree of job satisfaction was strongly and statistically significantly correlated with the degree of satisfaction from therapeutic relations (*r* = 0.535), emotional exhaustion (*r* = −0.400), and general life satisfaction (*r* = 0.256)—results not shown in detail.

### 4.4. Differences in the Frequency, Intensity, and Composite Moral Distress Scores by Sociodemographic and Work-Related Characteristics

Table 4 shows the observed differences in levels of moral distress score according to sociodemographic and work-related characteristics of the participants. These differences were assessed using the parametric *t*-test (comparisons between two independent groups of the samples) and one-way ANOVA test (comparisons among more than two independent groups of the sample) as appropriate. Table 4 also depicts correlations between all three moral distress scores (composite, intensity, frequency scores) and satisfaction variables, assessed with Pearson's *r* coefficient. There was a negative correlation between moral distress score and length of employment in the current position, which indicates that younger MHN with fewer years of experience are more likely to report higher levels of moral distress. Consistently, lower levels of moral distress were observed in terms of the age of the participants and their rank, with the lowest scores observed among participants over 40 years of age (*M* = 62.7 versus around 100 in all other age-groups; *p* < 0.001) and among senior nurses/managers (*M* = 52.3 versus 102.3 in nurses; *p* < 0.001). No other statistical significant differences were observed in moral distress scores by sociodemographic and work-related characteristics, including no differences in terms of the reported intention to quit, or staff safety measures.

### 4.5. Association of Moral Distress with Secondary Traumatic Stress Syndrome Symptoms and the Mediating Effect of Mental Distress Symptoms

Table 5 presents the association between composite moral distress score and STSS scale score as estimated in linear regression models before and after adjusting (a) for job satisfaction, satisfaction from therapeutic relations and emotional exhaustion (all important covariates), as well as (b) for general symptoms of mental distress as measured by the GHQ-28, which was found to be highly correlated with both the self-reported level of moral distress and the STSS scale score. There was a positive association between STSS scale score and composite moral distress score, after controlling for job satisfaction, satisfaction from therapeutic relations, and emotional exhaustion with a standardized beta coefficient of 0.269 SD increase in STSS scale score per SD increase in the moral distress composite score. After further adjusting for GHQ-28 score, the observed association attenuates slightly (st. beta = 0.154), suggesting that the association between moral distress and secondary traumatic stress syndrome symptoms is partly mediated by general symptoms of mental distress but remains statistically significant. No other study variables showed an association with STSS scale score. Specifically, the degree of empathy showed no association with the severity of STSS symptoms (Table 3) and there was no attenuation in the observed association after further controlling for empathy in the models (standardized beta = 0.159; *p* value = 0.005)—not shown in detail. The association also remained largely unchanged after adjusting for gender, age, number of children, education, rank, and intention to quit (standardized beta = 0.145; *p* value = 0.02). In fact, in a stepwise regression model with backward elimination including all study covariates, only moral distress composite score, GHQ-28 score, and emotional exhaustion score were predictive of STSS scale score, explaining 39.6% of the variance—results not shown in detail. Furthermore, while the association between moral distress composite score and STSS scale score appeared stronger in female MHN compared to male MHN (standardized beta = 0.181 versus 0.118), there was no evidence for effect modification (*p* for interaction = 0.77). Finally, there was no effect modification on the association between moral distress composite score and STSS score by age-group, education, rank, or intention to quit.

## 5. Discussion

For the first time, in a sample of mental health nurses in Europe, we assessed the intensity and severity of morally distressing situations, along with associations with the severity of secondary traumatic stress syndrome symptoms, and the mediating effect of general symptoms of mental distress. In contrast to previous investigations, we used an instrument addressing mental health practice both in the community and in mental health institutions, and we used a census sampling method to invite participation of all MHNs in Cyprus. We observed a positive association between the overall severity of moral distress and the manifestation of secondary traumatic stress syndrome symptoms, partially mediated by the degree of self-reported mental distress symptoms. Additionally, for the first time, we adjusted for a number of sociodemographic and work-related factors. These results are novel and add a new perspective to the so far limited data on the association between the severity of moral distress and secondary traumatic stress syndrome within the context of Mental Health Services [22, 66].

Yet, the contribution of the present data may be viewed under the light of lack of quantitative data regarding the intensity and severity of moral distress especially in mental health nurses in a European setting. Previous studies in this group of nurses either involved non-European populations, specifically Jordanian [44] and Japanese nurses, [60] and qualitative approaches [48, 67, 68], or have assessed concepts related to moral distress, for example, moral sensitivity, ethical climate, or ethical stress, as the severity of general stress responses related to moral dilemmas [69]. An additional contribution of this study is the use of an instrument specific for both hospital and community employed mental health nurses. In contrast, Ohnishi et al. [60] and Hamaideh [44] have used an instrument culturally oriented for hospital, mainly institutional, environments.

*Frequency and Intensity of MD. *The associations presented herein may be of particular significance for quality and safety improvement initiatives, since international literature signposts that health professionals' mental health status and work-related stress are of increasing concern due to their impact on staff and patient quality and safety standards [56, 70–72]. Data show that the vast majority of situations related to morally distressing experiences concern patient safety issues [67]. As a result, the frequency and intensity of morally distressing experiences among clinicians may be indicators of the quality and patient safety status predominant in a given healthcare setting [53]. Indeed, the top items with the highest morally distressing intensity and frequency reported herein regarded working with incompetent colleagues, application of unnecessary, extensive, and without consent therapeutic interventions, suspicions of patient abuse, and working under unsafe nurse-to-patient ratios. Thus, interventions oriented to both organizational culture and policies, with special focus on the particular conditions and needs prevailing in mental healthcare sector, are proposed. The importance of this approach is twofold. Firstly, morally distressing situations may be addressed and further managed having a direct, positive impact on patient safety standards. On the other hand, such interventions are important in mitigating work-related risks, mainly moral distress and related secondary traumatic stress syndrome symptoms, which may render nurses' vulnerable to severe mental and physiological health problems, with subsequent effects on patients' safety [73, 74].

In more detail, since moral distress has been associated with both mental distress and secondary traumatic stress symptoms herein, this may denote that fundamental neurocognitive functioning, such as cognitive procedures and perception, as well as emotionality, may be disrupted under morally distressing conditions [32]. Relevant mental disturbances, if not treated effectively, may influence MHNs professional performance within the work environment, including professional and therapeutic relations, along with communication issues, errors during medication treatment, patients' surveillance, and continuity of care [20, 31, 34]. The latter may be disrupted due to MHNs' high rates of sick leaves or turnover associated with diminished level of health [31, 33–35].

*Factors Associated with MD*. In relation to the factors linked with moral distress, although we have found that younger MHN with fewer years of work experience are more likely to experience higher levels of moral distress, and that one out of four participants reported intention to quit the job due to moral distress, there was no effect modification on the association between moral distress and secondary traumatic stress syndrome symptoms by age-group, rank, or intention to quit. This lack of modification effect may signify that the effects of moral distress are uniform across groups of MHN and that neither age nor longer work experience and no rank can protect nurses, who experience both mental and moral distress symptoms, from developing traumatic symptomatology. Furthermore, the association between moral distress and severity of secondary traumatic stress syndrome symptoms remained largely unchanged after adjusting for gender, number of children, and education, which may further denote the universal impact and profound effects of moral distress, regardless of the differences in one's context and presumably coping style.

Therefore, resilience to moral distress may not be easily attainable based on personal attributes or circumstances [75]. Indeed, a recent consensus committee on developing resilience to moral distress focused on policy, practice, and education [76]. Though experienced on a personal level, moral distress needs to be viewed as an organizational problem. Thus, effective interventions need to address the sources of moral distress within the social and organizational context of the workplace. Although resilience among clinicians may be an important buffering personality trait, it cannot be an effective antidote to moral distress, especially when the work environment is permissive to circumstances leading to moral distress.

Overall, organizational empowerment oriented interventions are proposed to allow MHNs to speak up and advocate effectively for the patients they care for, mainly within the multiprofessional therapeutic teams [67]. Special focus needs to be paid in younger and novice nurses, that is, younger than 40 years old, staff MHNs. It seems that probably due to the subculture of the organization or the setting of employment this group of nurses is more frequently reluctant to reveal information regarding morally distressing situations. This may be explained through the adaptation model of nursing [77]. Based on this model, MHNs may be viewed as adaptive, unique structures of interrelated systems, that is, biological, psychological, and social, which have inputs and outputs, along with an internal process aiming to maintain a balance between the three interrelated systems (biological, psychological, and social) and the outside environment, that is, the working environment of Mental Health Services. However, according to this model there is no absolute level of balance, while MHNs strive to adapt within a unique band in which they can cope adequately. Therefore, MHNs, in order to sustain the balance between their needs linked with the social system (acceptance from colleagues) and the work environment, may not reveal disturbing information about, for example, an unethical behaviour of a colleague or impeaching data for a healthcare organization regarding patient safety standards. On the other hand, although an adaptation process may be ongoing, it is possible an for an imbalance, between the system of MHNs' psychological needs and the ethical climate of the work environment, to occur, which may be reflected on disturbed mental health among MHNs, for example, anxiety and depressive symptoms.

Adaptation model in nursing may also be useful regarding the formation of targeted interventions aiming to decline both formal and informal organizational structures which encumber MHNs to advocate effectively for the patients, or even their colleagues. Relevant interventions may include augmentation of the degree of nurse managers' participation in committees related to both mental healthcare policy making and administration of Mental Health Services, therefore to support issues and decisions which enhance MHNs' structural empowerment, that is, access for all ranking staff nurses to resources and information. Furthermore, MHN managers need to support novice staff MHNs to speak up and sufficiently participate in clinical decision-making about direct patient care. Nevertheless, the acknowledgement of the input of novice staff nurses by nurse managers, both in private and within the interdisciplinary therapeutic team, may empower novice nurses to speak up and provide valuable information in relation to morally distressing issues linked with patient safety.

Additionally, nurses, and especially younger and less experienced nurses, need to be empowered organizationally in terms of effective collaboration with physicians and colleagues, as well as enhanced clinical and organizational autonomy [11]. Unit-based interventional programs aiming to enhance the quality of communication among colleagues, as well as with the members of multidisciplinary team [78], along with establishment of multidisciplinary rounds [79] may be useful towards this goal.

Nevertheless, MHNs need to be also personally empowered. This may be achieved through interventions which promote implementation of effective coping strategies towards work-related stress and assertiveness attitude, as well as in-unit education in the issues of moral distress. Assertiveness training is expected to enable MHNs to overcome the existing informal hierarchy within colleagues and interdisciplinary team in cases they perceive that their moral code is violated either by the rules and norms of the healthcare organization or by the decisions of other healthcare employees [80]. Education aiming to empower and enhance a critical clinical approach, along with engagement in continuing professional development, is also expected to increase the accountability of novice and low-ranking staff nurses, as well as to promote a productive collaboration with the rest of the members of the multidisciplinary team.

*Degree of Emotional Exhaustion, Job Satisfaction, and Empathy. *The participants herein reported a moderate degree of work satisfaction, empathy, and job-related emotional exhaustion. The latter was also a predictive of secondary traumatic stress syndrome symptoms. The severity of the emotional exhaustion level, along with the moderate levels of job satisfaction, may be explained on the basis of the recent reformation of Mental Health Services in Cyprus. During the last three decades, there has been a gradual move of therapeutic services from institutional (Athalassa hospital) to community settings. To date, community Mental Health Services are available to individuals and their families that experience distressing situations or chronic and acute mental health problems, across Cyprus [81]. Nevertheless, although the vast majority of Mental Health Service consumers visit community settings, the majority of MHNs are employed in institutional (Athalassa hospital) and hospital settings (psychiatric clinics in general hospitals). International data suggests that the reformation of Mental Health Services is followed by greater demands on community MHNs and can subsequently result in increased workload in conjunction with the greater complexity of the needs of the community Mental Health Service consumers [82]. Thus, the increased demands of the reformed professional role may have taken their toll on Cypriot MHN's work-related stress and feelings about their job [83]. Policy makers need to address these issues and intervene accordingly. For instance, postgraduate education in advanced clinical roles supported by the healthcare organization of MHNs' employment may empower MHNs to effectively manage the complex needs of Mental Health Service consumers. The aforementioned context may have also influenced the degree of empathy reported herein.

*Association among MD, STSS, Mental Distress, and Emotional Exhaustion*. The association among emotional exhaustion, moral distress, STSS, and mental distress may suggest that the recent reformation in the Mental Health Services and the subsequent increase in the workload of MHNs—implying high levels of emotional exhaustion—along with the current organizational, typical and atypical, norms existing may have bent MHNs more vulnerable to the development of both STSS and moral distress symptoms [51]. Data show that exposure to prolonged stress is associated with decrease in the plasticity of the brain and diminished production of the Brain Neurotrophic Factors [5, 84, 85]. This condition has been also associated with susceptibility to anxiety and depressive symptoms, as well as relevant manifestations, probably suggesting vulnerability to moral distress or STSS symptoms, as well [84, 85]. Based on this, further research regarding longitudinal studies on the association between work-related stress, biomarkers, and moral distress or STSS symptoms may be valuable. However, we need to state that this association denotes the opposite as well, that is, that those MHNs who experience symptoms of STSS or moral distress are more prone to develop professional burnout symptoms.

Additionally, the mediating effect of emotional exhaustion on the association between moral distress and STSS symptoms may be also explained on the basis of the information exchanged within the context of the therapeutic relation with patients [21, 51]. In more detail, many times the therapeutic communication encompasses patients' confessions about traumatic experiences, for example, being a victim or an offender of sexual or physical abuse [22]. As a result, MHNs may be influenced by this confession in terms of symptoms of secondary traumatic stress symptoms. At the same time, if they are not able to reveal relevant information or to advocate for the victims, for example, to activate legal procedures towards those involved in such circumstances, it is possible to experience symptoms of moral distress [51]. Moreover, the effective therapeutic management of these patients, as well as legal consequences of the perpetrators, may be also hindered due to inadequate law system or referral systems within Mental Health Services, lack of qualified mental health professionals, for example, forensic personnel, or even heavy workload and insufficient time for communication with patients [51–53, 63]. Such conditions may trigger not only morally distressing symptoms for the vulnerable personnel but emotional exhaustion, as well.

*The Mediating Effect of Mental Distress Level on the Association between MD and STSS. *The association between moral distress and symptom severity of secondary traumatic stress syndrome, partially mediated by the manifestation of symptoms of mental distress, may suggest that mental health nurses experiencing clinically relevant symptoms of mental distress are more prone to develop secondary traumatic stress symptoms related to morally distressing situations in their daily clinical practice. This finding denotes that the cost of unresolved moral distress may be high in nurses additionally experiencing mental distress symptoms. One possible explanation may be that those nurses who experience general symptoms of mental distress, mainly depressive ones, most of the times have decreased self-esteem. Thus, encountering morally distressing situations may further compromise one's personal integrity and self-esteem making him/her more vulnerable to further worsening of depressive and anxiety symptoms. In that case nurses may perceive that they abandon their moral principles due to fear, expediency, or self-protection, resulting in further decrease of their self-esteem and further worsening of preexistent anxiety or depressive symptoms [86, 87].

The aforementioned findings highlight the necessity for the development and implementation of supportive interventions for vulnerable groups of MHNs, such as those at higher risk to encounter morally distressing situations (e.g., employed in involuntary admission wards) [53]. Moreover, nurses already reporting mental distress symptoms need to be supported in cases they are faced with morally distressing situations in order to prevent deterioration. Counseling, along with supportive and educative clinical supervision, may be useful for this group [88, 89]. In Cyprus, there is a lack of that kind of professionals' support in place for MHNs, who may only seek such services privately. Nevertheless, the cost of counseling is high, thus not affordable to the vast majority of clinical MHNs. This lack of supportive systems in the Cypriot healthcare system needs to be addressed; therefore interventions implemented by healthcare managers and policy makers towards this goal are proposed. The aim is to incorporate within each organization officially provided supportive counseling services for mental health professionals.

Although moral distress may be a phenomenon attributed to workplace rather than to personal characteristics, raising awareness of moral distress and its effects may empower nurses and instigate organizational change. Thus, along with interventions regarding changes in the organizational culture and institutional framework of clinical MHNs, it would be advisable to enrich the curricula of nursing and other health professionals educational programmes with work-related stress topics, including burnout, moral distress, and vicarious trauma giving special emphasis to preventive strategies along with education on effective coping skills and leadership against these phenomena.

According to Austin et al. [68] “although the term, moral distress, is not part of our ordinary language, using it can help us speak to the moral domain of our practice.” When individuals are informed about a topic, they are also able to name relevant experiences, which may lead them to a higher level of awareness [90]. When perceptions and feelings are put into words people are empowered to act upon them. Naming and understanding the distress that arises when nurses are blocked from responding to the needs of their patients are the first step towards empowering themselves to action [68]. Overall, the debate about ethics in clinical settings can be productive and positive, a sign that healthcare providers are engaged in collaborative relationships and concerned about the quality of care for their patients [91].

Furthermore, nursing managers need to be aware of these phenomena. Nevertheless, experiences of moral distress are further compounded when clinicians perceive managers and administrators as not being adequately open or supportive during morally challenging situations [92].

Our results may also suggest that optimal mental health status in MHNs functions as a buffer system against the impact of morally distressing or trauma situations experienced within the work context. Based on this, interventions supporting mental health in MHNs may decrease their susceptibility to the negative impact of prolonged work-related stress. Recently, holistic interventions, such as S&R (a program providing healthy snacks and holistic relaxation modalities), have been implemented for healthcare employees, aiming to alleviate immediate feelings of work-related stress [73]. These interventions have been associated with decrease in self-reported stress, respiration, and heart rate. In conclusion, consistent provision of occupational health services within workplaces needs to be incorporated as a vital component of the public health strategy.

### 5.1. Limitations

The present findings need to be regarded in the context of specific methodological limitations. The cross-sectional design of the study does not permit causal inferences with regard to the direction of the relationship between professional moral distress and symptoms of secondary traumatic stress syndrome [61]. While the observed association is strong, reverse causality cannot be ruled out. MHN with secondary traumatic stress syndrome symptomatology may be more likely to experience or at least report more intense moral distress. Similarly, while the study showed that the association between moral distress and secondary traumatic stress syndrome symptoms was mediated by symptoms of mental distress, it is also likely that mental distress (which could be the result of a number of non-work-related situations) is also a cofounder in this association; that is, MHN with mental distress symptoms may be more likely to experience moral distress as a result. While the study focused on work-related sources of distress, it is also likely that other life stressors beyond work tension are at play. Nevertheless, the association between moral distress and secondary traumatic stress syndrome symptoms remained unchanged after controlling for satisfaction with social and general personal life, which was reported to be quite high in contrast to work satisfaction and work-related emotional exhaustion. Another issue related to the limitations of the present study is the use of the M-MDS-MHS instrument for the assessment of moral distress, developed for the purpose of the present study [51]. Although its development was based on the items of previous instruments [60, 62], showing adequate metric properties, its content and construct validity need to be further explored. A particular strength of the study is the inclusion of nurses from diverse work settings, as well as the fact that the entire nursing population employed in Mental Health Services was approached. Furthermore, the composition of the sample is representative of the expected distribution of the workforce employed in Cypriot Mental Health Services, according to official data. Thus, the findings may be generalized to the nursing populations of all mental healthcare settings. Nevertheless, the extent to which selection bias may have influenced the observed association, due to the voluntary nature of participation and the lower response rate, is not known. It is possible that nurses suffering from severe mental and/or moral distress symptoms were less likely to participate. Thus, we may have underestimated the actual frequency and severity of relevant symptoms, which nevertheless appear quite high.

*Future Perspectives for Research. *Future prospective studies need to investigate work-related factors that may lead to both moral distress and symptoms of mental distress, including secondary traumatic stress syndrome. Also, both qualitative and quantitative studies, exploring moral distress experiences, taking into account gender inequalities and power relations within the healthcare systems, or even adaptation model in nursing, may provide valuable data. Moreover, future researchers aiming to develop instruments for the measurement of moral distress in healthcare professionals, mainly employed in Mental Health Services, need to include issues relevant to secondary traumatic stress factors, as well as to increase the inclusion of factors related to workload and emotional exhaustion factors.

## 6. Conclusions

Our findings provide preliminary evidence of the association between moral distress and secondary traumatic stress symptoms in MHN. Since secondary traumatic stress syndrome symptoms were more likely among mental health nurses who experienced morally distressing situations, and general symptoms of mental distress, supportive interventions are warranted. Situations that may lead health professionals to be in moral distress seem to be mainly related to the work environment; thus interventions related to organizational empowerment of MHNs need to be developed.

## Figures and Tables

**Table 1:** Sociodemographic or work-related characteristics of the participants and self-reported satisfaction measures (*N* = 206).

Variable			
	Categories	Frequency (*N* = 206)	Relative frequency (%)

Gender	Female	116	56.3%
	Male	90	43.7%

Age	<30	48	23.3%
	30–34	71	34.5%
	35–39	40	19.4%
	≥40	43	20.9%
	Not reported	4	1.9%

Education	2- or 3-year diploma	4	1.9%
	Bachelor degree	126	61.2%
	Postgraduate degree	73	35.4%
	Doctoral degree	1	0.5%
	Not reported	2	1.0%

Marital status	Married/cohabiting	135	66.2%
	Single	54	26.5%
	Divorced, separated, widowed	15	7.4%

Children	None	73	35.4%
	One	45	21.8%
	Two	63	30.6%
	Three or more	25	12.2%

Physical activity	Never	78	37.9%
	1-2 times/week	76	37.3%
	More than 3 times/week	50	24.5%
	Not reported	2	1%

Alcohol use	Not weekly	110	53.4%
	1-2 glasses/week	87	42.2%
	More than 3/week	4	1.9%
	Not reported	5	2.4%

Region	Nicosia	119	57.8%
	Limassol	56	27.2%
	Larnaca	18	7.8%
	Paphos/Famagusta	15	7.3%

Setting	Athalassa psychiatric hospital	45	22.1%
	Psychiatric wards in general hospitals	20	9.8%
	Inpatient substance use treatment units	15	7.4%
	Inpatient child/adolescent units	14	6.8%
	Community mental health services	49	24.0%
	Psychosocial rehabilitation units	7	3.4%
	Outpatient child/adolescent services	17	8.3%
	Outpatient substance use treatment units	31	15.2%
	Mental health services in Prison	6	2.9%

Position	Nurse	180	87.8%
	Senior Nurse	23	11.2%
	Nurse Manager	2	1.0%

Intention to leave	Have previously left position	3	1.5%
	Considered leaving position	43	20.9%
	Never considered leaving	146	70.9%
	Not reported	14	6.8%
		Mean (SD)	Median (IQR)

Overall work experience (years)		11.6 (7.4)	10.0 (6–15)
Length of employment in current position (years)		4.0 (3.8)	3.0 (1–t5)
Job satisfaction (NAS 0–10)		6.6 (2.1)	7.0 (5–8)
General personal life satisfaction (NAS 0–10)		8.0 (1.5)	8.0 (7–9)
Satisfaction with social relationships (NAS 0–10)		7.9 (1.4)	8.0 (7–9)
Satisfaction with therapeutic relations (NAS 0–10)		7.3 (1.8)	8.0 (7–8.5)
Degree of emotional exhaustion (NAS 0–10)		4.8 (2.7)	5.0 (3–7)

**Table 2:** Summary statistics (*N* = 206), Cronbach's alpha coefficient of internal consistency (*N* = 206), and test-retest correlation coefficient for main study variables (overall scale and/or subscales).

Instrument overall scale	Subscale (number of items, theoretical range)	Mean (SD)	Median (IQR)	Range	Cronbach's alpha coefficient	Test-retest Pearson's *r* (*p*-value)
Modified version of Moral Distress Scale for Mental Health Services (M-MDS-MHS)	Frequency, 32 items (theoretical range: 0–128)	*37.8 (20.1)*	*38 (24–51)*	*1–103*	*α* = 0.962	*r* = 0.645 (*p* < 0.001)
	Intensity, 32 items (theoretical range: 0–128)	*76.4 (31.1)*	*71 (47–97)*	*0–127*	*α* = 0.926	*r* = 0.793 (*p* < 0.001)
	Composite score, multiplicative (theoretical range: 0–512)	*96.8 (62.9)*	*91 (51–135)*	*0–345*		

Secondary Traumatic Stress Scale	17 items (theoretical range: 17–85)	*31.1 (10.2)*	*30 (24–37)*	*17–61*	*α* = 0.925	*r* = 0.848 (*p* < 0.001)

Jefferson Scale of Empathy	20 items (theoretical range: 20–140)	*105.9 (12.7)*	*106 (100–115)*	*69–134*	*α* = 0.777	*r* = 0.897 (*p* = 0.006)

General Health Questionnaire-28 (GHQ-28)	Somatic symptoms, 7 items (theoretical range: 0–21)	*5.8 (3.5)*	*5 (3–8)*	*0–18*	*α* = 0.855	*r* = 0.755 (*p* < 0.001)
	Anxiety/insomnia, 7 items (theoretical range: 0–21)	*5.4 (3.9)*	*5 (3–7)*	*0–18*	*α* = 0.898	*r* = 0.751 (*p* < 0.001)
	Social dysfunction, 7 items (theoretical range: 0–21)	*6.7 (2.6)*	*7 (6-7)*	*0–17*	*α* = 0.837	*r* = 0.680 (*p* = 0.001)
	Depression, 7 items (theoretical range: 0–21)	*5.0 (2.2)*	*1 (0–2)*	*0–12*	*α* = 0.830	*r* = 0.845 (*p* < 0.001)
	Overall, 28 items (theoretical range: 0–84)	*19.4 (10.0)*	*17 (13–24)*	*0–55*		

**Table 3:** Pearson's correlation coefficients (*p* values) between main study variables (*N* = 206).

	M-MDS-MHS, overall	M-MDS-MHS, frequency	M-MDS-MHS, intensity	SPTS Scale	Jefferson Scale of Empathy	GHQ-28, overall	GHQ, somatic symptoms	GHQ, anxiety/insomnia symptoms	GHQ, social/personal dysfunction	GHQ, depressive symptoms
M-MDS-MHS, Composite	1.00									
M-MDS-MHS, Frequency	0.91^*∗∗∗*^	1.00								
M-MDS-MHS, Intensity	0.53^*∗∗∗*^	0.33^*∗∗∗*^	1.00							
STSS Scale	0.35^*∗∗∗*^	0.37^*∗∗∗*^	0.16^*∗∗*^	1.00						
Jefferson Scale of Empathy	0.035	−0.13	0.18^*∗∗*^	−0.10	1.00					
GHQ-28, overall	0.29^*∗∗∗*^	0.27^*∗∗∗*^	0.19^*∗∗*^	0.65^*∗∗∗*^	−0.10	1.00				
GHQ, somatic symptoms	0.31^*∗∗∗*^	0.28^*∗∗∗*^	0.20^*∗∗*^	0.52^*∗∗∗*^	−0.07	0.89^*∗∗∗*^	1.00			
GHQ, anxiety/insomnia symptoms	0.31^*∗∗∗*^	0.30^*∗∗∗*^	0.22^*∗∗*^	0.70^*∗∗∗*^	−0.09	0.91^*∗∗∗*^	0.79^*∗∗∗*^	1.00		
GHQ, personal/social dysfunction	0.11	0.07	0.11	0.37^*∗∗∗*^	−0.01	0.68^*∗∗∗*^	0.49^*∗∗∗*^	0.46^*∗∗∗*^	1.00	
GHQ, depressive symptoms	0.14^*∗*^	0.18^*∗*^	0.05	0.43^*∗∗∗*^	−0.21^*∗∗*^	0.70^*∗∗∗*^	0.49^*∗∗∗*^	0.55^*∗∗∗*^	0.33^*∗∗∗*^	1.00

^*∗*^ *p* value < 0.05, ^*∗∗*^*p* value < 0.01, and ^*∗∗∗*^*p* value < 0.001.

**Table 4:** Differences in the frequency, intensity, and composite moral distress score by sociodemographic and work-related characteristics.

Variable	Categories	Moral distress, frequency Mean (SD)	Moral distress, intensity Mean (SD)	Moral distress, composite score Mean (SD)
Gender	Female	40.1 (21.2)	68.0	97.8 (62.9)
	Male	36.0 (19.1)	72.3	96.3 (63.4)
	*p* value^†^	0.15	0.35	0.90

Age	<30	42.2 (21.2)	70.2 (30.5)	107.7 (68.0)
	30–34	40.2 (18.6)	70.5 (28.8)	102.1 (54.2)
	35–39	39.5 (17.9)	75.6 (32.8)	106.2 (56.2)
	≥40	26.1 (19.1)	62.8 (38.0)	62.7 (63.8)
	*p* value^†^	<0.001	0.34	0.001

Education	Diploma/Bachelor degree	37.4 (20.4)	69.2 (31.6)	93.7 (63.0)
	Postgraduate/Doctoral	38.7 (19.1)	73.2 (32.4)	102.0 (61.3)
	*p* value^†^	0.65	0.38	0.36

Marital status	Married/cohabiting	42.9 (21.2)	73.6 (29.4)	109.1 (66.3)
	Single	35.8 (19.2)	68.3 (32.6)	91.2 (59.7)
	Divorced, separated, widowed	31.9 (18.5)	73.8 (39.0)	83.7 (62.7)
	*p* value^†^	0.53	0.05	0.15

Children	None	42.4 (21.3)	74.1 (29.2)	108.4 (67.4)
	One	35.4 (19.9)	69.5 (34.9)	92.0 (61.4)
	Two	37.5 (17.8)	67.8 (32.3)	95.2 (53.4)
	Three or more	29.8 (21.1)	68.1 (35.5)	75.2 (70.6)
	*p* value^†^	0.04	0.67	0.12

Physical activity	Never	40.6 (19.9)	74.1 (28.3)	104.8 (62.7)
	1-2 times/week	33.1 (19.4)	66.8 (37.4)	86.1 (64.7)
	More than 3 times/week	41.7 (20.2)	72.0 (27.9)	103.8 (58.6)
	*p* value^†^	0.02	0.35	0.13

Alcohol use	Not weekly	35.7 (20.4)	68.0 (34.9)	90.8 (63.5)
	1-2 glasses/week	40.6 (19.6)	74.2 (28.4)	106.0 (62.5)
	More than 3/week	51 (23.8)	71.3 (15.4)	121.0 (50.5)
	*p* value^†^	0.10	0.41	0.19

Position	Nurse	39.6 (19.7)	73.3 (30.4)	102.3 (61.7)
	Senior nurse/manager	23.6 (16.7)	48.4 (36.5)	52.4 (50.6)
	*p* value^†^	<0.001	<0.001	<0.001

Intention to leave	Have left/considered leaving	37.1 (19.7)	70.2 (32.7)	100.5 (65.2)
	Never considered leaving	38.1 (20.0)	72.5 (31.7)	97.6 (62.8)
	*p* value^†^	0.77	0.67	0.79

Variable		Pearson's correlation coefficient (*p* value)		

Overall work experience (years)		−0.17 (0.02)	−0.01 (0.86)	−0.12 (0.08)
Length of employment in current position (years)		−0.14 (0.05)	−0.08 (0.27)	−0.15 (0.04)
Job satisfaction (NAS 0–10)		−0.14 (0.05)	−0.09 (0.21)	−0.15 (0.03)
General personal life satisfaction (NAS 0–10)		0.02 (0.78)	0.17 (0.02)	0.11 (0.14)
Satisfaction from social relationships (NAS 0–10)		−0.02 (0.77)	0.05 (0.45)	0.01 (0.99)
Satisfaction from therapeutic relations (NAS 0–10)		−0.21 (<0.01)	−0.23 (<0.01)	−0.21 (<0.01)
Degree of emotional exhaustion (NAS 0–10)		0.10 (0.14)	0.13 (0.07)	0.19 (<0.01)

^†^ *p* value of independent *t*-test or one-way ANOVA as appropriate.

**Table 5:** Association of secondary traumatic syndrome symptoms with moral distress before (model 1) and after adjusting for work-related satisfaction covariates (model 2) and mental distress level (model 3).

	Secondary Traumatic Stress Scale score											
	Univariable model (model 1)				After adjusting for satisfaction and emotional exhaustion covariates (model 2)				Also, adjusting for GHQ-28 score (model 3)			
	*B*	St. error	St. beta	*p* value	*b*	St. error	St. beta	*p* value	*b*	St. error	St. beta	*p* value
Moral distress composite score	0.056	0.011	**0.345**	<0.001	0.044	0.011	**0.269**	<0.001	0.025	0.009	**0.154**	0.005
Job satisfaction level					0.049	0.393	0.010	0,90	−0.023	0.326	−0.005	0.94
Satisfaction from therapeutic relations					−0.761	0.421	−0.135	0.07	−0.383	0.352	−0.068	0.28
Emotional exhaustion level					1.021	0.262	0.268	<0.001	0.386	0.227	0.101	0.09
GHQ-28 score									0.565	0.059	0.551	<0.001

	*F* = 27.896, *p* < 0.001, adjusted *R*^2^ = 0.12				*F* = 13.833, *p* < 0.001, adjusted *R*^2^ = 0.20				*F* = 34.422, *p* < 0.001, adjusted *R*^2^ = 0.45			
